# Polyhydroxylated fullerene nanoparticles attenuate brain infarction and oxidative stress in rat model of ischemic stroke

**DOI:** 10.17179/excli2016-309

**Published:** 2016-06-20

**Authors:** Javad Rasouli Vani, Mohammad Taghi Mohammadi, Mahsa Sarami Foroshani, Mahvash Jafari

**Affiliations:** 1 Department of Nanotechnology, School of New Sciences and Technology, Islamic Azad University of Pharmaceutical Sciences Branch, Tehran, Iran; 2 Department of Physiology and Biophysics, School of Medicine, Baqiyatallah University of Medical Sciences, Tehran, Iran; 3 Department of Biochemistry, School of Medicine, Baqiyatallah University of Medical Sciences, Tehran, Iran

**Keywords:** ischemic stroke, oxidative damage, nitrosative damage, fullerene nanoparticles

## Abstract

Oxidative stress is the common underlying mechanism of damage in ischemic stroke. Therefore, we aimed to evaluate the possible protective effects of polyhydroxylated fullerene derivatives on brain infarction and oxidative/nitrosative stress in a rat model of ischemic stroke. The experiment was performed by four groups of rats (each; n=12); Sham, Control ischemia, and ischemic treatment groups (Pretreatment and Posttreatment). Brain ischemia was induced by 90 min middle cerebral artery occlusion (MCAO) followed by 24 hours reperfusion. Rats received fullerene nanoparticles at dose of 1 mg/kg 30 min before MCAO and immediately after beginning of reperfusion. Infarct volume, contents of malondialdehyde (MDA), glutathione (GSH) and nitrate as well as superoxide dismutase (SOD) activity were assessed 24 hours after termination of MCAO. Brain infarct volume was 310 ± 21 mm^3^ in control group. Administration of fullerene nanoparticles before and after MCAO significantly decreased the infarct volume by 53 % (145 ± 45 mm^3^) and 81 % (59 ± 13 mm^3^), respectively. Ischemia also enhanced MDA and nitrate contents of ischemic hemispheres by 45 % and 25 % , respectively. Fullerene nanoparticles considerably reduced the MDA and nitrate contents of ischemic hemispheres before MCAO by 58 % and 17 % , respectively, and after MCAO by 38 % and 21 % , respectively. Induction of MCAO significantly decreased GSH content (19 % ) and SOD activity (52 % ) of ischemic hemispheres, whereas fullerene nanoparticles increased the GSH content and SOD activity of ischemic hemispheres by 19 % and 52 % before MCAO, respectively, and 21 % and 55 % after MCAO, respectively. Our findings indicate that fullerene nanoparticles, as a potent scavenger of free radicals, protect the brain cells against ischemia/reperfusion injury and inhibit brain oxidative/nitrosative damage.

## Introduction

Oxidative stress is the common underlying mechanism of damage in ischemic stroke (Niizuma et al., 2010[33]; Chen et al., 2011[6]; Rodrigo et al., 2013[36]). It has been demonstrated that free radicals lead to injuries in various cellular components including proteins, lipids and DNA (Gilgun-Sherki et al., 2002[11]; Slemmer et al., 2008[41]; Kleinschnitz et al., 2010[20]). Previous findings have shown that about 2-5 % of the electron flow in isolated mitochondria from brain tissue generates reactive oxygen species (ROS), (Bioveris and Chance, 1973[1]). These continually generated ROS such as hydrogen peroxide (H_2_O_2_) and superoxide anion (O_2_**^-^**) are quenched by the brain antioxidant enzymes such as superoxide dismutase (SOD), glutathione peroxidase (GSHPx) and catalase (CAT), (Slemmer et al., 2008[41]; Gill and Tuteja, 2010[12]). On the other hand, these antioxidant enzymes are inactivated when free radicals are overproduced by activation of pro-oxidant enzymes during brain ischemia (Kinouchi et al., 1991[19]). Many pro-oxidant enzymes participate in free radicals generation at ischemic brain such as xanthine oxidase (XO), nitric oxide synthase (NOS) and NADPH-oxidase (Chan, 2001[5]; Miller et al., 2006[27]; Ishizuka et al., 2008[16]). Based on previous findings, upregulation of NADPH-oxidase is the main endogenous source of ROS during brain ischemia (Kehrer, 2000[17]; Yoshioka et al., 2011[48]). Nitric oxide (NO) is another poisonous free radical that is overproduced by different NOS isoforms during brain ischemia (Han et al., 2006[13]; Mohammadi et al., 2011[31]; Mohammadi and Dehghani, 2015[30]). Overproduction of NO in ischemic brain induces nitrosative damage (Parathath et al., 2006[34]; Mohammadi et al., 2012[32]). Ultimately, combination of NO and ROS results in formation of very toxic compound of peroxynitrite (ONOO^-^), which yields to the protein nitrotyrosination and cell death (Kehrer, 2000[17]). 

Recently, fullerene (C_60_) nanoparticles, which are the third allotrope of carbon atoms in spherical structure, have been presented as the powerful antioxidants in biological systems (Lens et al., 2008[21]; Markovic and Trajkovic, 2008[26]; Liao et al., 2010[23]). They can easily react with electrons and abolish different free radicals (Chistyakov et al., 2013[7]). Based on recent findings, these nanoparticles are able to scavenge various free radicals more efficiently than cellular antioxidants (Cai et al., 2008[4]; Chistyakov et al., 2013[7]). Since fullerene nanoparticles are not soluble in biological environments, they must be water-soluble by adding the functional groups such as carboxyl (COOH) or hydroxyl (OH) (Injac et al., 2013[15]). Fullerene hydroxylation, which widely used as a scavenger in plentiful research, is suitable for this purpose (Kim et al., 2008[18]; Chistyakov et al., 2013[7]). Deguchi et al*.* (2006[9]) showed the antioxidant activities of hydroxyl fullerenes in aqueous environments. Kim et al. (2008[18]) demonstrated that hydroxyl fullerenes are able to protect neurons against transient global cerebral injury in rat hippocampus. Zha et al. (2012[49]) reported that fullerenol in cultured hippocampal neurons protects against cell damage and promotes cell viability mainly due to its influence on reduction-oxidation pathways. Additionally, intracerebroventricular administration of fullerene (0.3 mg/kg) had a protective effect against ischemia/reperfusion injury (Lin et al., 2002[24]). Finally, Ye et al. (2014[46]) reported that polyhydroxylated fullerene attenuates oxidative stress-induced apoptosis via a fortifying Nrf2-regulated cellular antioxidant system.

According to previous findings that water-soluble fullerene derivatives have free radical scavenging properties, we aimed to examine the possible protective effects of polyhydroxylated fullerene derivatives (C_60_(OH)_18-22_) against ischemia/reperfusion-induced brain damage in rat model of ischemic stroke. Additionally, we analyzed the possible protective role of this compound against oxidative or nitrosative damage, which induced by brain ischemia.

## Materials and Methods

### Animals

Male Wistar rats, 10-12 weeks old (280-320 g), were purchased from the animal house facility of Baqiyatallah University of Medical Sciences. The protocols of present study, which followed the NIH Guidelines for animals use and care, were approved by the institutional animal ethics committee of Baqiyatallah University of Medical Sciences. Rats were kept in the separate cages in a room with controlled temperature (22-24° C), light period (07.00-19.00), and humidity (40-60 % ). All animals access to rat chow and water *ad libitum*.

### Middle cerebral artery (MCA) occlusion 

Animals had been fasted overnight prior to use without deprivation of water. The rats were anesthetized with 2.5 % isoflorane (Forane, UK) and placed in dorsal recumbent position. Core temperature was maintained at 37 ± 1° C during the experiment using with a heating pad and continuous recording by a rectal probe connected to a thermistor. 

**Figure 1:**
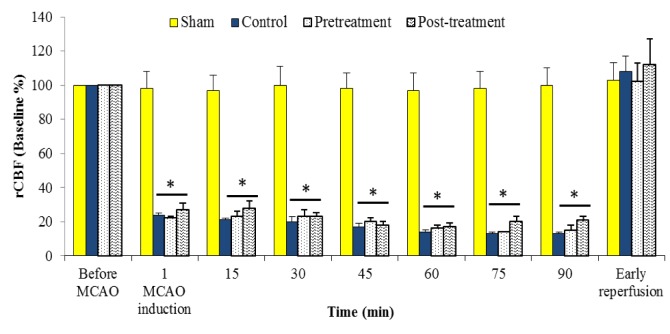
Regional cerebral blood flow (rCBF; % from baseline) before and during MCAO, and in the early reperfusion period in Sham, Control, Pretreatment and Posttreatment groups. All values are mean ± SEM. * as significant difference compared with Sham (P < 0.05)

Intraluminal filament method was used for middle cerebral artery occlusion (MCAO) of the right brain hemisphere to achieve brain ischemia (Longa et al., 1989[25]). “In brief, the right common carotid artery was exposed through a midline neck incision, and then, via external carotid artery, a 4-cm Poly-L-Lysine-coated nylon thread (3-0) was inserted into the internal carotid artery and gently advanced up until feeling a resistance and seeing a sharp decline in the blood flow trace. MCAO was maintained for 90 min, and then the thread was gently taken out to reestablish blood flow to the ischemic region. Finally, all the incisions were sutured, the animals were allowed to recover from anesthesia, and returned to a warm cage for recuperation during reperfusion period. Regional cerebral blood flow (rCBF) for the area of the right hemisphere that was nourished by the right middle cerebral artery (MCA) was recorded using a laser Doppler flowmeter (AD Instrument, Model: ML191, Australia). There was a 75-85 % reduction in rCBF of ischemic groups during MCA occlusion (Figure 1(Fig. 1)). This reduction returned swiftly back to its pre-occluded level during the first 15 min of reperfusion period” (Mohammadi and Dehghani, 2015[30]).

### Experimental protocols and groups

In **Sham** rats (n=12), the animals underwent the surgery at the neck area and received a single intraperitoneal injection of the 1 mL/kg normal saline, as vehicle, without being exposed to MCA occlusion. Surgery was performed at the neck region of control ischemic rats (**Control**, n=12) same as sham group. These animals received a single intraperitoneal injection of the 1 mL/kg normal saline, as vehicle, 30 min before MCA occlusion. After a 15 min rest, brain ischemia was performed by 90 min MCAO followed by 24 hours reperfusion. The rats of the ischemic pretreatment group (**Pretreatment**, n=12) received a single *i.p.* injection of 1 mg/kg polyhydroxylated fullerenes (Sigma, Germany) in 1 mL normal saline 30 min before induction of MCAO and other procedures were followed same as control group. The ischemic posttreatment group (**Posttreatment**, n=12) received a single *i.p.* injection of 1 mg/kg polyhydroxylated fullerenes in 1 mL normal saline immediately after termination of MCAO and beginning of reperfusion. Other protocols were followed the same as control group.

The number of rats presented for each group is the number of animals that survived during twenty four hours reperfusion period. The collected data of the rats that died in twenty four hours reperfusion period were excluded. The percent of mortality in control ischemia, pretreatment and posttreatment groups was 38 %, 13 % and 9 %, respectively. 

### Neurological assessment

Neurological functions were assessed at the beginning and twenty four hours of reperfusion in animals that survived the ischemic trauma and at same time periods in the sham rats. “A five-point grading scale of neurological deficit scores (NDS) was used, in which rats with normal motor function or no observable neurological deficits were assigned as grade 1. Grade 2 was given to rats that showed flexion of contralateral torso or forelimb upon lifting by their tail, or failure to extend their forepaw when suspended vertically, forelimb flexion and shoulder adduction. Grade 3 was for rats circling to the contralateral side of the MCA occluded hemisphere when the animal is held by the tail on a flat surface, but with normal posture at rest. Grade 4 was assigned to the loss of righting reflex and decreased resistance to lateral push, and finally, grade 5 was for no spontaneous motor activity” (Mohammadi et al., 2012[32]).

### Evaluation of brain infarction

Brain infarction was assessed according to the 2, 3, 5-triphenyltetrazolium chloride (TTC, Sigma) staining method. “In brief, after induction of deep anesthesia with sodium thiopental, the animals were slaughtered. Then, their brains were removed, cleaned, and solidified by immersing in pre-cooled normal saline (4^° ^C) and keeping in the refrigerator for 5 min. The prepared slices were stained with 2 % TTC and fixed in 10 % buffered formalin solution. After staining, the color of the ischemic areas was white and of non-ischemic areas was red. The slice images were digitized by using a Cannon camera. Images of the stained sections were taken. Grossly visible infarction zones were quantified using image analysis software (NIH Image Analyzer) and finally cerebral infarct volume was calculated as described previously” (Sarshoori et al., 2014[38]).

### Tissue swelling

Twenty four hours after reperfusion the rats were killed and the brains were removed. The brain divided into two hemispheres. Olfactory bulb and brain stem were removed and determined their total volume of hemispheres based on infarct volume method (Swanson et al., 1990[43]). Tissue swelling percentage was calculated using the following formula:

% Tissue swelling = [(V_Right Hemisphere_-V_Left Hemishpere_) / V_Left Hemisphere_] x 100

### Tissue preparation

After deep anesthesia, brains were quickly removed, washed in an ice-cold phosphate buffer saline (PBS) for assessment of malondialdehyde (MDA), glutathione (GSH) and nitrate contents as well as superoxide dismutase (SOD) activity. Tissues were immediately immersed in liquid nitrogen and kept on -80° C until biochemical analysis. On the day of use, the frozen samples were weighed and homogenized 1:10 in ice-cold PBS. The homogenates were then centrifuged at 14000×g for 15 min at 4° C. The supernatants were separated and used for measurement of GSH, MDA, nitrate and protein concentrations as well as SOD activity.

### Determination of glutathione (GSH) content 

The content of glutathione (GSH) was measured using the Tietz method (Tietz, 1969[44]). Cellular protein was precipitated by addition of 5 % sulfosalicylic acid and removed by centrifugation at 2000 g for 10 min. GSH in the supernatant was assayed as follows: 100 µL of the protein-free supernatant of the cell lysate, 800 µL of 0.3 mM Na_2_HPO_4_ and 100 µL of 0.04 % 5,5′-dithiobis-(2-nitrobenzoic acid) (DTNB) in 0.1 % sodium citrate. The absorbance of DTNB was monitored at 412 nm for 5 minutes. A standard curve of GSH was performed and sensitivity of measurement was determined to be between 1 and 100 µM. The level of GSH was expressed as µg/mg protein” (Mohammadi et al., 2013[29]).

### Assessment of SOD activity

The SOD activity was determined using the method described by Winterbourn et al. (1975[45]) based on the ability of SOD to inhibit the reduction of nitroblue tetrazolium (NBT) by superoxide. “For assay, 0.067 M potassium phosphate buffer, pH 7.8 was added to 0.1 M EDTA containing 0.3 mM sodium cyanide, 1.5 mM NBT and 0.1 mL of sample. Then, 0.12 mM riboflavin was added to each sample to initiate the reaction and was incubated for 12 min. The absorbance of samples was read on a Genesys 10 UV spectrophotometer at 560 nm for 5 minutes. The amount of enzyme required to produce 50 % inhibition was taken as 1 U and results were expressed as U/mg protein” (Mohammadi et al., 2013[29]).

### Determination of MDA as an index of lipid peroxidation

The end product of lipid peroxidation was estimated by measuring the level of MDA according to the Satoh method (Satoh, 1978[39]). “0.5 mL of tissue homogenate was added to 1.5 mL of 10 % trichloroacetic acid (TCA), vortexed and incubated for 10 min at room temperature. 1.5 mL of supernatant and 2 mL of thiobarbituric acid (0.67 % ) were added and placed in a boiling water bath in sealed tubes for 30 min. The samples were allowed to cool at room temperature. 1.25 mL of n-butanol was added, vortexed and centrifuged at 2000 g for 5 min. The resulting supernatant was removed and measured at 532 nm on a spectrophotometer. MDA concentrations were determined by using 1,1,3,3-tetraethoxypropane as standard. Finally, the MDA concentration was expressed as µg/mg protein” (Mohammadi et al., 2013[29]).

### Nitrate assay

The nitrate content of the brain was measured by the colorimetric reaction of the Griess reagent (Riahi et al., 2016[35]). 0.1 mL of homogenate solution was deproteinized by adding 0.2 mL of zinc sulfate solution and then centrifuged for 20 min at 4000 g and 4° C to separate supernatant for nitrate assay. 0.1 mL of supernatant (as sample) or pure water (as blank) or sodium nitrite (as standard) was mixed with 0.1 mL vanadium III chloride to reduce nitrite to nitrate. 0.05 mL sulfanilamide (0.01 % ) and 0.05 mL N-[1-naphthyl] ethylenediamindihydrochloride (NED, 0.01 % ) were incubated for 30 min in dark place at 37° C. Thereafter, the absorbance of solution was determined at wave length of 540 nm. Nitrate concentration was estimated from a standard curve generated from absorbance of each sodium nitrate solution. Finally, the nitrate level was expressed as µg/mg protein.

### Assessment of protein concentration

The concentration of protein was assessed according to the Bradford method using bovine serum albumin (BSA) as a standard (Bradford, 1976[3]).

### Statistical analysis

All values are presented as mean ± SEM. The comparison of data between groups were done by analysis of variance (ANOVA) followed by Tukey post-hoc test. The Mann-Whitney U test was used to analyze the NDS. For all states, *P *< 0.05 was considered as a significant difference.

## Results

### Regional cerebral blood flow (rCBF)

Representative changes of rCBF (% from baseline) with time are shown in Figure 1(Fig. 1). There was no significant difference in the rCBF of sham rats with time. Whereas, MCAO drastically reduced rCBF to about 15-25 % of their own baselines in ischemic groups (control, pretreatment and posttreatment). Results of rCBF for the ischemic rats indicated that treatments of ischemic animals with polyhydroxylated fullerenes did not statistically change the blood flow of the ischemic areas during ischemia or in the early reperfusion period.

### Neurological deficit score (NDS)

**Figure 2:**
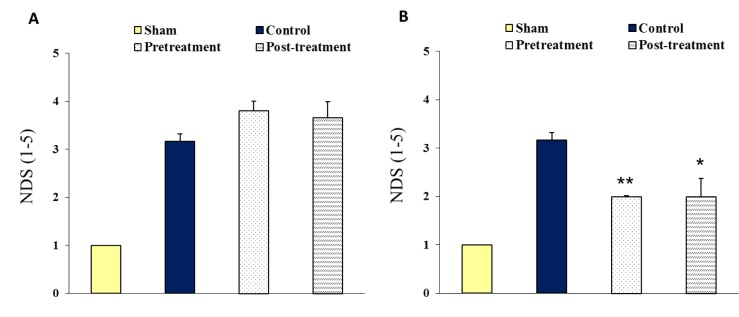
Neurological deficit score (NDS). (A): one hour after neck surgery in Sham or after 90 min MCAO in ischemic groups (Control, Pretreatment and Posttreatment), (B): 24 hours after neck surgery in Sham or after 90 min MCAO and 24 hours reperfusion in ischemic groups. All values are as Mean ± SEM. * as significant difference compared with Control (P < 0.05); ** as significant difference compared with Control (P < 0.01)

Figure 2(Fig. 2) represents the neurological disabilities (NDS) of ischemic groups at the beginning (A) and 24 hours (B) of reperfusion. The graph A is indicating that there were severe motor disabilities in ischemic groups (control, pretreatment and posttreatment) after occlusion of MCA. There was no significant difference in the values of NDS between ischemic groups following one hour reperfusion. Pretreatment and posttreatment with polyhydroxylated fullerenes showed a significant improvement of neurological disabilities following 24 hours reperfusion (graph B). 

### Brain infarction 

**Figure 3:**
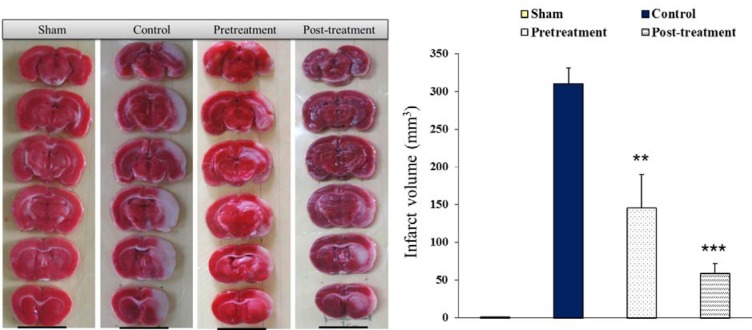
Photograph (left hand) is showing the coronal sections of rat's brain stained with triphenyltetrazolium chloride (TTC) 24 hours after neck surgery in Sham or after 90 min MCAO and 24 hours reperfusion in Control, Pretreatment and Posttreatment groups. Non-ischemic areas are colored deep red, whereas ischemic areas are white (Scale bar; 1 cm). The graph (right hand) is showing the infarct volume (mm^3^) for mentioned groups at the same time. All values are as Mean ± SEM. * as significant difference compared with Control (P < 0.01); ** as significant difference compared with Control (P < 0.001)

Qualitative evaluation of brain infarction is performed by the preparation of coronal sections stained with TTC (Figure 3(Fig. 3), left hand). The uniform red color of the sections of right and left hemispheres in sham rats indicated that the surgery did not induce infarction. The presence of white color areas intermingled with red color regions in the right hemispheres of ischemic rats (control, pretreatment and posttreatment) indicated that 90 min right MCAO provoked different magnitudes of brain infarctions without affecting the left hemispheres. The comparisons of the white color areas of the lesioned hemispheres of ischemic rats indicated that polyhydroxylated fullerenes attenuated the infarct size in both treated groups. Quantification of the ischemic areas of lesioned hemispheres indicated that MCA occlusion considerably induced brain infarction (310 ± 21 mm^3^). Pretreatment and posttreatment with polyhydroxylated fullerenes significantly attenuated the brain infarction in both treated groups (145 ± 45 mm^3^ and 59 ± 13 mm^3^, respectively), (Figure 3(Fig. 3), right hand).

### Tissue swelling of the ischemic hemispheres 

**Figure 4:**
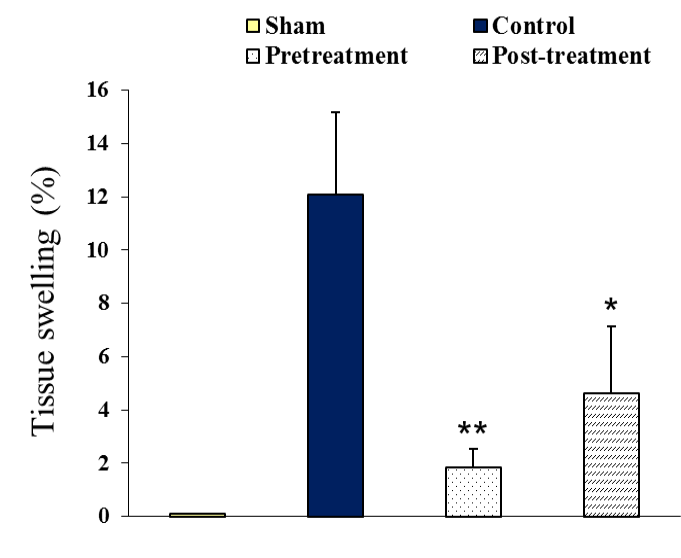
Tissue swelling (%) of right hemispheres 24 hours after neck surgery in Sham or after 90 min MCAO followed by 24 hours reperfusion in ischemic groups (Control, Pretreatment and Posttreatment). All values are as Mean ± SEM. * as significant difference compared with Control (P < 0.05); ** as significant difference compared with Control (P < 0.01)

The calculated percentage of tissue swelling (brain swelling) of MCA occluded hemispheres (right hemisphere) are presented in Figure 4(Fig. 4). The value of brain swelling in sham group was zero. Occlusion of MCA induced tissue swelling (12.08 ± 0.08 % ) following 24 hours reperfusion in control ischemic rats, whereas there was an 84 % and 61 % reduction in brain swelling in pretreatment (1.84 ± 0.69 % ) and posttreatment (4.63 ± 2.52 % ) groups, respectively. 

### SOD activity and GSH content of ischemic hemispheres 

**Figure 5:**
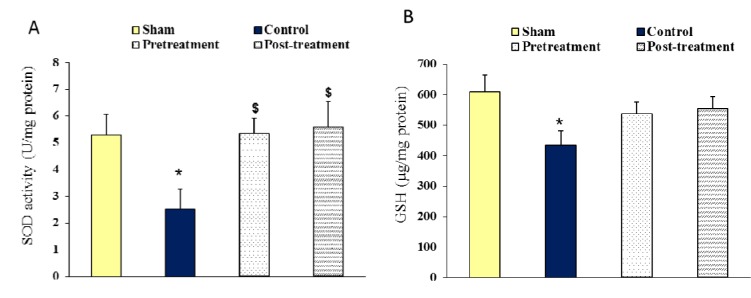
A; The activity of superoxide dismutase (SOD, U/mg protein), and B; Glutathione (GSH) content (µg/mg protein), of right hemispheres in Sham group 24 hours after neck surgery or after 90 min MCAO and 24 hours reperfusion in Control, Pretreatment and Posttreatment groups. All values are as Mean ± SEM. * as significant difference compared with Sham (P < 0.05); ^$^ as significant difference compared with Control (P < 0.05)

Figure 5(Fig. 5) denotes the relative SOD activity (A) and GSH content (B) of ischemic hemispheres (right hemispheres) after 90 min MCA occlusion followed by 24 hours reperfusion. The results of ischemic brain are indicating that occlusion of MCA decreased the SOD activity of control ischemic group (2.52 ± 0.75 U/mg protein) compared to sham (5.30 ± 0.76 U/mg protein). Ischemia also decreased the GSH content of ischemic group (434 ± 47 µg/mg protein) compared with sham (610 ± 54 µg/mg protein). Treated rats with polyhydroxylated fullerenes showed a significant increment of SOD activity in both pretreatment (5.35 ± 0.57 U/mg protein) and posttreatment (5.59 ± 0.96 U/mg protein) groups. Furthermore, the total GSH content of ischemic hemispheres increased in both pretreatment (536 ± 39 µg/mg protein) and posttreatment (553 ± 41 µg/mg protein) groups but these changes did not statistically differ compared to control group. 

### MDA and nitrite contents of ischemic hemispheres

**Figure 6:**
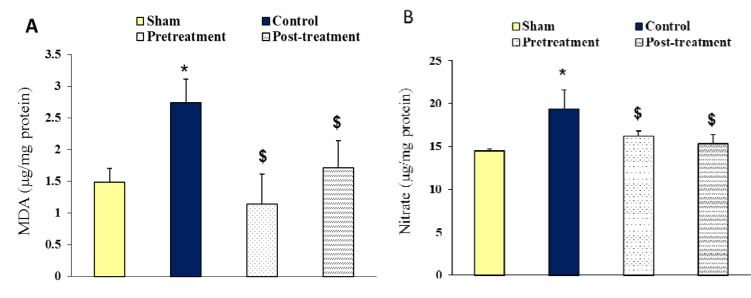
A; Representative changes of malondialdehyde (MDA) content (µg/mg protein), and B; nitrate content (µg/mg protein), of right hemispheres in Sham group 24 hours after neck surgery or after 90 min MCAO and 24 hours reperfusion in Control, Pretreatment and Posttreatment groups. All values are as Mean ± SEM. * as significant difference compared with Sham (P < 0.05); ^$^ as significant difference compared with Control (P < 0.05)

Figure 6(Fig. 6) shows the MDA (A) and nitrate (B) contents of ischemic hemispheres (right hemispheres) after 90 min MCA occlusion followed by 24 hours reperfusion. The results of ischemic brains indicate that occlusion of MCA increased the MDA content of control group (2.74 ± 0.37 µg/mg protein) compared to sham (1.49 ± 0.22 µg/mg protein). Ischemia also enhanced the nitrate content of control group (19.41 ± 02.20 µg/mg protein) compared to the sham (14.50 ± 0.25 µg/mg protein). Treated rats with polyhydroxylated fullerenes showed a significant reduction of MDA in both treated groups following 24 hours reperfusion (pretreatment; 1.14 ± 0.48 µg/mg protein, posttreatment; 1.71 ± 0.43 µg/mg protein). Furthermore, the nitrate content of ischemic hemispheres decreased in both pretreatment (16.18 ± 0.65 µg/mg protein) and posttreatment (15.32 ± 1.02 µg/mg protein) groups compared to control. 

## Discussion

Recently, several nanoparticles have been introduced for a wide variety of biological applications (Cai et al., 2008[4]; Chistyakov et al., 2013[7]). Previous studies have shown that water-soluble fullerene derivatives are able to quench all of the major physiologically relevant ROS like superoxide anion and protect the cells against oxidative damages (Lens et al., 2008[21]; Markovic and Trajkovic, 2008[26]; Liao et al., 2010[23]). In the present study, we indicated the protective effects of polyhydroxylated fullerene nanoparticles (C_60_(OH)_18-22_) against brain ischemia/reperfusion injury in rat. Using a rat model of transient MCA occlusion, we showed the protective effects of these nanoparticles on brain infarction. Our findings revealed a decrease in oxidative/nitrosative damage of ischemic brain by fullerene nanoparticles. Also, these nanoparticles potentiated the brain antioxidant system of ischemic treated rats compared to ischemic untreated animals. It is suggested that fullerene nanoparticles, as a powerful scavenger of free radicals, protect the brain cells against ischemia/reperfusion-induced brain damage.

In the present study, intraperitoneal administration of polyhydroxylated fullerenes (1 mg/kg) significantly decreased the neurological score (NDS), infarct volume and tissue swelling of ischemic hemispheres in both treated groups (pretreatment and posttreatment). Our findings indicate that polyhydroxylated fullerenes during ischemia or reperfusion phase are able to improve the injuries of ischemic stroke. Based on previous studies, functionalized fullerenes interfere in processes of the ischemic cascades such as inflammation, oxidative stress, apoptotic cascades and other neurotoxic pathways, which play critical roles in pathophysiology of ischemic stroke (Markovic and Trajkovic, 2008[26]; Zha et al., 2012[49]; Fluri et al., 2015[10]). Fluri et al. (2015[10]) demonstrated the anti-inflammatory properties of functionalized fullerenes following brain ischemia. In other studies, Yin et al. (2009[47]) and Markovic and Trajkovic (2008[26]) reported the ability of fullerene derivatives to quench various free radicals and behave as a free radical sponge. Also, the study of Hu et al. (2010[14]) indicated that water-soluble fullerene derivatives have the potential to prevent NO-mediated cell death without evident toxicity. All of the previous reports confirm that water-soluble fullerenes have a great ability to protect the different cells against apoptosis, particularly neurons. Furthermore, the protective effects of water-soluble fullerene derivatives against ischemia/reperfusion injuries have been reported in other tissues such as intestine and in other in vitro organ perfusate (Chueh et al., 1999[8]; Bisaglia et al., 2000[2]). These observations indicate the potential therapeutic properties of the functionalized fullerene derivatives to prevent the injuries of ischemic stroke.

The findings of present study indicated that brain contents of MDA (an index of oxidative damage) and nitrate (an index of nitrosative damage) considerably enhanced after induction of brain ischemia/reperfusion. A large number of in vitro and in vivo studies suggest that ROS and nitrogen reactive species (RNS) are involved in pathophysiology of brain ischemia/reperfusion injuries (Niizuma et al., 2010[33]; Chen et al., 2011[6]; Rodrigo et al., 2013[36]). These free radicals interact with various biomolecules of cells resulting in lipid peroxidation, DNA damage, protein modification and alterations in enzymatic activities, ultimately leading to apoptotic or necrotic cell death (Gilgun-Sherki et al., 2002[11]; Slemmer et al., 2008[41]; Kleinschnitz et al., 2010[20]). Our results indicated that using polyhydroxylated fullerenes before or after MCA occlusion greatly decreased the contents of brain MDA and nitrate. It is well known that water-soluble fullerene derivatives possess a great capacity for scavenging ROS and RNS (Markovic and Trajkovic, 2008[26]; Hu et al., 2010[14]). Yin et al. (2009[47]) reported the scavenging of ROS and the potential for cell protection by functionalized fullerene materials. Markovic and Trajkovic (2008[26]) demonstrated that fullerene nanoparticles have a potential ability to quench ROS, behaving as a ''free radical sponge''. Likewise, it has been demonstrated that polyhydroxylated fullerene (C_60_(OH)_24_) was able to quench NO and block its biological activity in vivo (Hu et al., 2010[14]). According to the study of Misirkic et al. (2009[28]) fullerene nanoparticles might be candidate for preventing NO-mediated cell injury in inflammatory or autoimmune disorders. Finally, the capability of polyhydroxylated fullerene derivatives acting as ROS or RNS scavenger has been shown to be involved in inhibition of apoptosis in different types of cells both in vitro and in vivo (Bisaglia et al., 2000[2]; Hu et al., 2010[14]).

Our results indicate that the activity of SOD and total GSH content of brain have decreased in ischemic hemispheres. SOD is an important antioxidant enzyme of brain that quenches the superoxide anion in mitochondrial matrix (Rowley and Patel, 2013[37]). Based on previous findings, a decrease in the SOD level during brain ischemia promotes the neural death and brain damages (Slemmer et al., 2008[41]). Likewise, GSH is an intracellular element of brain cells that plays a crucial role in cellular protection against oxidant damage during brain ischemia (Song et al., 2015[42]). A decrease in cellular GSH content following brain ischemia induces the release of cytochrome c, which initiates the apoptotic signaling cascades (Schafer and Buettner, 2001[40]). In the present study, using polyhydroxylated fullerenes in ischemic rats increased the activity of SOD and total GSH content of ischemic brain. It is suggested that increased the SOD activity and total GSH content of brain in ischemic treated rats by polyhydroxylated fullerenes correlates with reduction of oxidative damage and brain infarction. Song et al. (2015[42]) reported that GSH might improve the pathogenesis of ischemic stroke by attenuating brain infarction and cell death. In addition, it is reported that the increment of SOD activity during brain ischemia ameliorates oxidative damage and improve ischemia/reperfusion-induced brain damages (Li et al., 2015[22]). However, other studies need to elucidate the direct effects of polyhydroxylated fullerenes on the activities of brain antioxidant enzymes and brain GSH in ischemic stroke.

In conclusion, the findings of present study indicate that polyhydroxylated fullerene nanoparticles (C_60_(OH)_18-22_) are able to improve the brain ischemia/reperfusion-induced neuronal death and brain damages. Based on our findings, polyhydroxylated fullerenes inhibit the oxidative and nitrosative damages of the ischemic brain that may be related to improving the brain antioxidant system. 

## Acknowledgements

The authors are cordially appreciating the financial support of Vice Chancellor for Research of Baqiyatallah University of Medical Sciences, Tehran, Iran. We also thank Dr. David Mc Clyment for critically editing this manuscript.

## Conflict of interest

The authors declare no conflict of interest.
